# Structural Determinants of the APOBEC3G N-Terminal Domain for HIV-1 RNA Association

**DOI:** 10.3389/fcimb.2019.00129

**Published:** 2019-05-21

**Authors:** Hirofumi Fukuda, Songling Li, Luca Sardo, Jessica L. Smith, Kazuo Yamashita, Anamaria D. Sarca, Kotaro Shirakawa, Daron M. Standley, Akifumi Takaori-Kondo, Taisuke Izumi

**Affiliations:** ^1^Department of Hematology and Oncology, Graduate School of Medicine, Kyoto University, Kyoto, Japan; ^2^Systems Immunology Laboratory, WPI Research Center Immunology Frontier Research Center, Osaka University, Osaka, Japan; ^3^Department of Genome Informatics, Genome Information Research Center, Research Institute for Microbial Diseases, Osaka University, Osaka, Japan; ^4^Department of Biological Sciences, McNeil Science and Technology Center, University of the Sciences, Philadelphia, PA, United States; ^5^Molecular and Translational Sciences, United States Army Medical Research Institute of Infectious Diseases, Frederick, MD, United States

**Keywords:** APOBEC3G, RNA, DNA, HIV-1 Vif, Structural Model, Imaging

## Abstract

APOBEC3G (A3G) is a cellular protein that inhibits HIV-1 infection through virion incorporation. The interaction of the A3G N-terminal domain (NTD) with RNA is essential for A3G incorporation in the HIV-1 virion. The interaction between A3G-NTD and RNA is not completely understood. The A3G-NTD is also recognized by HIV-1 Viral infectivity factor (Vif) and A3G-Vif binding leads to A3G degradation. Therefore, the A3G-Vif interaction is a target for the development of antiviral therapies that block HIV-1 replication. However, targeting the A3G-Vif interactions could disrupt the A3G-RNA interactions that are required for A3G's antiviral activity. To better understand A3G-RNA binding, we generated *in silic*o docking models to simulate the RNA-binding propensity of A3G-NTD. We simulated the A3G-NTD residues with high RNA-binding propensity, experimentally validated our prediction by testing A3G-NTD mutations, and identified structural determinants of A3G-RNA binding. In addition, we found a novel amino acid residue, I26 responsible for RNA interaction. The new structural insights provided here will facilitate the design of pharmaceuticals that inhibit A3G-Vif interactions without negatively impacting A3G-RNA interactions.

## Introduction

APOBEC3G (A3G), a member of the cellular cytidine deaminase APOBEC3 superfamily, exhibits anti-HIV activity primarily by inducing G-to-A hypermutation in the viral cDNA (Wedekind et al., 2003; Conticello et al., 2007; Conticello, 2008; Desimmie et al., 2016). The interaction of A3G with the HIV-1 nucleocapsid (NC) domain of Gag is necessary for A3G encapsidation into HIV-1 virions in virus producing cells (Luo et al., 2004; Schäfer et al., 2004; Svarovskaia et al., 2004; Zennou et al., 2004; Khan et al., 2005; Wang et al., 2007). A3G deaminates nascent cDNA strands generated by reverse transcription and potently restricts HIV-1 replication in target cells. HIV-1 Viral infectivity factor (Vif) counteracts the A3G-mediated antiviral activity by inducing degradation of A3G through the ubiquitin-proteasome pathway (Harris and Liddament, 2004; Izumi et al., 2008, 2009).

A3G requires RNA to interact with the NC domain of Gag for its encapsidation in the virion (Luo et al., 2004; Schäfer et al., 2004; Svarovskaia et al., 2004; Zennou et al., 2004). It has been shown that several RNAs, including the HIV-1 genomic RNA, the cellular Y RNAs, and 7SL RNA participate in these interactions (Khan et al., 2005; Wang et al., 2007). However, the structural basis of these interactions is controversial (Bach et al., 2008; Izumi et al., 2013; Apolonia et al., 2015). A3G has two homologous domains (Jarmuz et al., 2002; Wedekind et al., 2003; Navarro et al., 2005; Larue et al., 2009). The N-terminal domain (A3G-NTD), consisting of residues 1-200, is catalytically inactive but indispensable for Vif-interaction and A3G encapsidation (Bogerd et al., 2004; Mangeat et al., 2004; Schrofelbauer et al., 2004; Xu et al., 2004; Navarro et al., 2005; Iwatani et al., 2006; Huthoff and Malim, 2007; Izumi et al., 2010; Feng and Chelico, 2011; Bélanger and Langlois, 2015) and is also involved in the association with viral and cellular RNAs (Wang et al., 2007; Bach et al., 2008; Bulliard et al., 2009; Friew et al., 2009; Huthoff et al., 2009; Chelico et al., 2010; Lavens et al., 2010; Zhang et al., 2010; Shlyakhtenko et al., 2011; Uyttendaele et al., 2012; Apolonia et al., 2015; Bélanger and Langlois, 2015). Several groups have shown that amino acids 22-136 of the A3G-NTD are required for A3G-RNA interaction, A3G encapsidation, and A3G oligomerization (Huthoff et al., 2009; Bélanger and Langlois, 2015). Specifically, two tryptophan residues at position 94 and 127, and residues R24, S28, R30, R122, Y124, and F126 have been determined to be involved in A3G-RNA interactions (Table 1) (Huthoff and Malim, 2007; Bach et al., 2008; Bulliard et al., 2009; Huthoff et al., 2009; Zhang et al., 2010; Bélanger and Langlois, 2015).

**Table 1:**
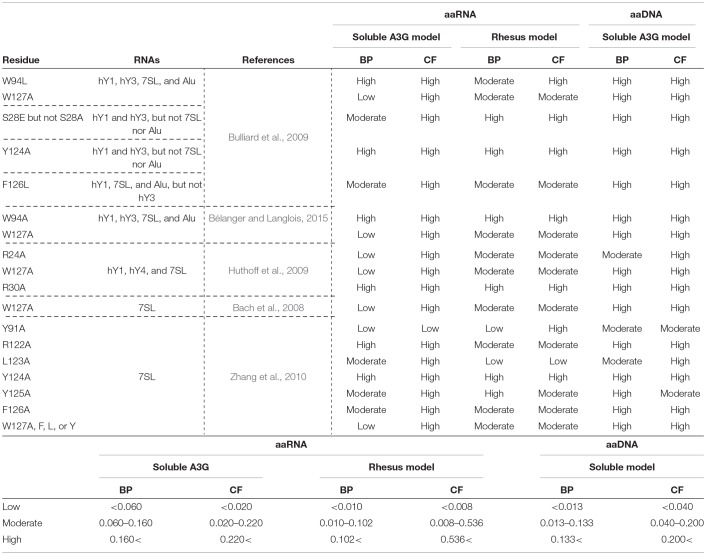
Amino acid residues in A3G-NTD reportedly involved in RNA association and their binding propensity (BP) and contact frequency (CF) of RNA or DNA as determined in our study.

Little is known about the molecular structure of the A3G-NTD and RNA complex. HIV-1 Vif mediates A3G degradation via the ubiquitin-proteasomal pathway (Sheehy et al., 2002; Harris et al., 2003; Mangeat et al., 2003; Zhang et al., 2003). Importantly, the A3G-Vif interaction is a valuable target for the design of novel antiviral molecules. In this regard, some of the previously identified A3G-RNA interacting residues, Y124 or W127 (Huthoff and Malim, 2007; Huthoff et al., 2009), are also necessary for the interaction of A3G with HIV-1 Vif, suggesting that the A3G-NTD binding surfaces for RNA and Vif may overlap (Uyttendaele et al., 2012). Therefore, better defining the A3G-RNA binding sites will aid the rational design of A3G-Vif inhibitors that do not exert off target effects on A3G-RNA binding.

Recently, Kouno et al. revealed the human A3G-NTD structure by solution NMR. For this purpose, they engineered a soluble form of the human A3G-NTD by introducing several mutations and deletions, resulting in a sequence identity of 80% with the wild-type (WT). Interestingly, this soluble A3G-NTD mutant was not packaged into virus particles (Kouno et al., 2015), suggesting that it could no longer interact with the target RNA. However, because of the considerably high sequence identity, the NMR structures are a useful tool for *in silico* modeling studies. Here, to account for A3G flexibility in simulation of RNA binding, we used a novel approach by generating an *in silico* A3G-RNA docking model based on ten A3G-NTD NMR structure snapshots. Further, we validated the accuracy of our model *in vitro* and *ex vivo* using full-length A3G alanine mutation analysis. In addition, we developed a second homology model based on the non-human primate A3G-NTD crystal structure (Xiao et al., 2016), and predicted its RNA docking patterns. These docking models mostly provided similar RNA association parameters and allowed us to identify A3G I26 as a novel residue involved in A3G-RNA association.

## Materials and Methods

### Plasmid Construction and Cell Culture

We constructed an expression vector of hemagglutinin (HA)-tagged human A3G, pcDNA3/HA-A3G, as previously described (Kobayashi et al., 2004) that we used for single site A3G mutations (Y22E, I26A, S28A, R29A, R30A, Y86A, R122A, Y124A, and E259Q) generated with the QuickChange XL site directed mutagenesis kit (Stratagene). The C-terminal EYFP-tagged A3G expression plasmids were generated by inserting the above mentioned A3G fragments amplified by PCR into the NheI and KpnI site of pEYFP-N1 vector (Clontech). A 3xFLAG synthesized DNA was inserted between the A3G and EYFP coding regions (pA3G-3xFLAG-EYFP). For visualizing virus particles, we used an HIV-1 based construct that expresses the fusion protein Gag containing the mCherry fluorescent protein with HIV-1 protease recognition sequence between MA and CA (imCH) as previously reported (Hübner et al., 2007). A stop codon was inserted into the *vif* region and the *env* gene was frame-shifted to be deleted in the imCH vector (imCHΔVifΔEnv). Adherent HEK293T cells or non-adherent M8166 cells were cultured in 10% Fetal Calf Serum of Dulbecco's Modified Eagle's Medium or RPMI Medium, respectively (Kobayashi et al., 2004). Cells were maintained at 37°C with 5% CO_2_.

### Molecular Modeling of the A3G N-Terminal Domain

#### Homology Modeling

The original amino acid sequence of human A3G-NTD (1–200) was aligned to either the soluble form of human A3G-NTD (PDBID: 2mzz) (Kouno et al., 2015) or the crystal structure of a non-human primate A3G (PDBID: 5k81) (Xiao et al., 2016) and rendered in 3D by Spanner (Lis et al., 2011). Ten NMR structures and one crystal structure were used for model building followed by RNA-binding site prediction with the aaRNA algorithm (Li et al., 2014) or DNA-binding site prediction with the aaDNA algorithm.

#### RNA Docking Simulations

The ESPResSo (Limbach et al., 2006) molecular dynamics package was used for all coarse-grained molecular dynamics (CGMD) simulations. To simplify the model, we represented each amino acid and nucleotide residue as single-beads and fixed each protein structure during the simulation. A soft core potential was introduced between protein and nucleotides so that the nucleotide could not enter the core region of the protein. The binding propensity (BP) of each amino acid was used as an additional contact potential to sample reliable RNA or DNA binding conformations. While the RNA BP was predicted by a counterpart network model based on RNA-binding proteins, the DNA BP was predicted by an artificial neural network model based on DNA-binding proteins. For each soluble A3G model, we randomly distributed 100 non-specific 5-mer RNA or DNA molecules around the protein to initialize the system. A total of 10,000 snapshots were stored for each soluble model and grouped by progressive clustering with 10Å as a threshold. The clusters were sorted from higher to lower scores, and the top five clusters were investigated further. The contact frequency (CF) of each amino acid was calculated based on the top five clusters from each soluble model. For the single crystal A3G model, a total of 100 clusters were used for CF calculation of each amino acid.

#### Statistical Analysis of BP and CF

Because both the BP and CF of each amino acid do not follow normal distributions, we calculated the average, standard deviation (SD), median, and quartiles of the all BP and CF. High values were defined as being greater than the third quartile, while low values were defined as being less than the first quartile.

### Single-Virion Imaging Analysis

To visualize the encapsidation of EYFP-tagged A3G or its mutants in virions, pA3G-3xFLAG-EYFP was co-transfected with imCHΔVifΔEnv in HEK293T cells (3.5 million per 10 cm dish) with the PEI transfection reagent (GE Healthcare). The virus-containing supernatant was harvested at 24 h post-transfection, filtered through 0.45 μm pore size sterile cellulose membrane and concentrated up to 20-fold by ultracentrifugation through a 20% sucrose cushion at 25,000 rpm for 90 min at 4°C (CP65; Hitachi Koki Co., Ltd.). The concentrated virus supernatant (0.5 μL) was briefly mixed with 200 μL Hank's Balanced Salt Solution (HBSS) (+) without Phenol Red (Wako) and mounted onto 8-well glass bottom chamber slides (Matsunami), then incubated overnight at 4°C. Images of virions were acquired with an A1R Confocal Microscope (Nikon). mCherry was used to identify virus particles and EYFP was used to detect incorporated A3G protein. Images were first exported using the NIS-Elements software (Nikon) and representative images were processed with Fiji, an image processing package in ImageJ (NIH). A binary image of the mCherry and EYFP signals was generated to count the positive puncta. A3G encapsidation efficiency was calculated as percentage of mCherry positive signals that co-localized with the EYFP signal within a 3-pixel range from the centroid of the mCherry signal by an in-house MATLAB program (MathWorks).

### Immunoblotting and Fluorescence Measurement

Cells were lysed using RIPA buffer (Wako) supplemented with protease inhibitor cocktail (Sigma-Aldrich) and the supernatants were collected after centrifugation. Polyacrylamide gel electrophoresis and protein transfer to PVDF membranes (Immobilon, Millipore) were followed by hybridization with primary antibodies. Blots were probed with rabbit anti-HA (Sigma-Aldrich), mouse anti-Vif (NIH AIDS Reagent Program), and mouse anti-GFP (Thermo Fisher Scientific) primary antibodies overnight at 4°C. HRP-conjugated anti-mouse or rabbit IgG antibodies (GE Healthcare) were used as secondary antibodies. Western blot images were obtained with x-ray films. EYFP fluorescence intensity in the cell lysates was measured with a plate reader (Perkin Elmer, 2030 ARVO X3).

### qPCR Assay

A3G and its derived mutants (Y22E and R122A) were immunoprecipitated with anti-HA antibody conjugated Protein A beads from cell lysates extracted from transfected HEK293T cells. After immunoprecipitation, A3G associated RNAs were purified with the High Pure RNA extraction kit (Roche). The concentration of purified RNA was measured by a NanoDrop ND-1000 Spectrophotometer and adjusted to the total RNA amount prior to the reverse transcriptase (RT) reaction. The RT product was obtained by TaKaRa PrimeScript 1st strand cDNA Synthesis Kit. The RNA denaturing process was performed at 65°C for 5 min, followed by 4°C for 10 min with 1 μL Random hexamers and 1 μL dNTP mix. The denatured RNAs were then reverse-transcribed with 4 μL 5xPrimeScript Buffer, 20 units RNase Inhibitor (40 U/μL), 200 units PrimeScript RTase, and 4.5 μL RNase free water. The RT reaction was performed at 30°C for 10 min, 42°C for 60 min, 95°C for 5 min and then 4°C overnight. A Real-Time PCR instrument, Thermal Cycler Dice Real Time System TP800 (TaKaRa) was used for monitoring PCR amplifications. All primers were synthesized by SIGMA. qRT-PCR was performed using SYBR green. Each reaction mixture contained 0.5 μL of forward and reverse primers (10 μM), 10 μL of THUNDERBIRD SYBR qPCR Mix (TOYOBO), 7 μL of RNase-free water, and 2 μL of template cDNA. The reactions were performed under the following conditions: 94.0°C for 10 s, followed by 40 cycles of 95°C for 5 s and 60°C for 30 s, for the final step. Single peaks in the melting-curve analysis indicated specific amplicons. Primer sequences used in these assays were previously published (Wang et al., 2007). Each Y and 7SL RNA association to A3G was compared to the mock sample whose level was set to 1. Three independent experiments were performed. *P*-values were calculated relatively to the A3G WT.

### Escherichia Coli Mutation Assay

The BW310 strain of *E. coli* was transformed with pTrc-His A based Isopropyl β-D-1-thiogalactopyranoside (IPTG)-inducible A3G expression vectors or the empty vector (Petersen-Mahrt et al., 2002). Individual colonies were picked and grown to saturation in LB medium containing 50 μg/mL ampicillin and 1 mM IPTG. Appropriate dilutions were spread onto agar plates containing either 50 μg/mL ampicillin or 100 μg/mL rifampicin and incubated overnight at 37°C. Mutation frequencies were recorded as the number of rifampicin-resistant colonies per 10^6^ viable cells, which were enumerated using the ampicillin containing plates. Colony counts were recorded in this manner for 6 rifampicin- and 2 ampicilin-containing plates for each construct. The average colony count for A3G WT was set to 100% and all other scores were normalized to this value.

### Single-Round Infection Assay

The VSV-G pseudotyped HIV-1-ΔVif luciferase reporter viruses with A3G or its mutants were produced by co-transfection of pNL4-3/ΔenvΔvif-Luc and pVSV-G with the A3G expression vector (pcDNA3/HA-A3G) in HEK293T cells as previously described (Shindo et al., 2003; Kobayashi et al., 2004). The target M8166 cells were infected with reporter viruses normalized by p24 amount, which was measured by HIV Type 1 p24 Antigen ELISA (ZeptoMetrix). The infectivity was measured by luciferase activity and values were normalized to the infectivity of the virus produced in the absence of A3G expression.

## Results

### Homology Model Structure Based Calculation of the A3G-RNA Binding Propensity

**Figure 1:**
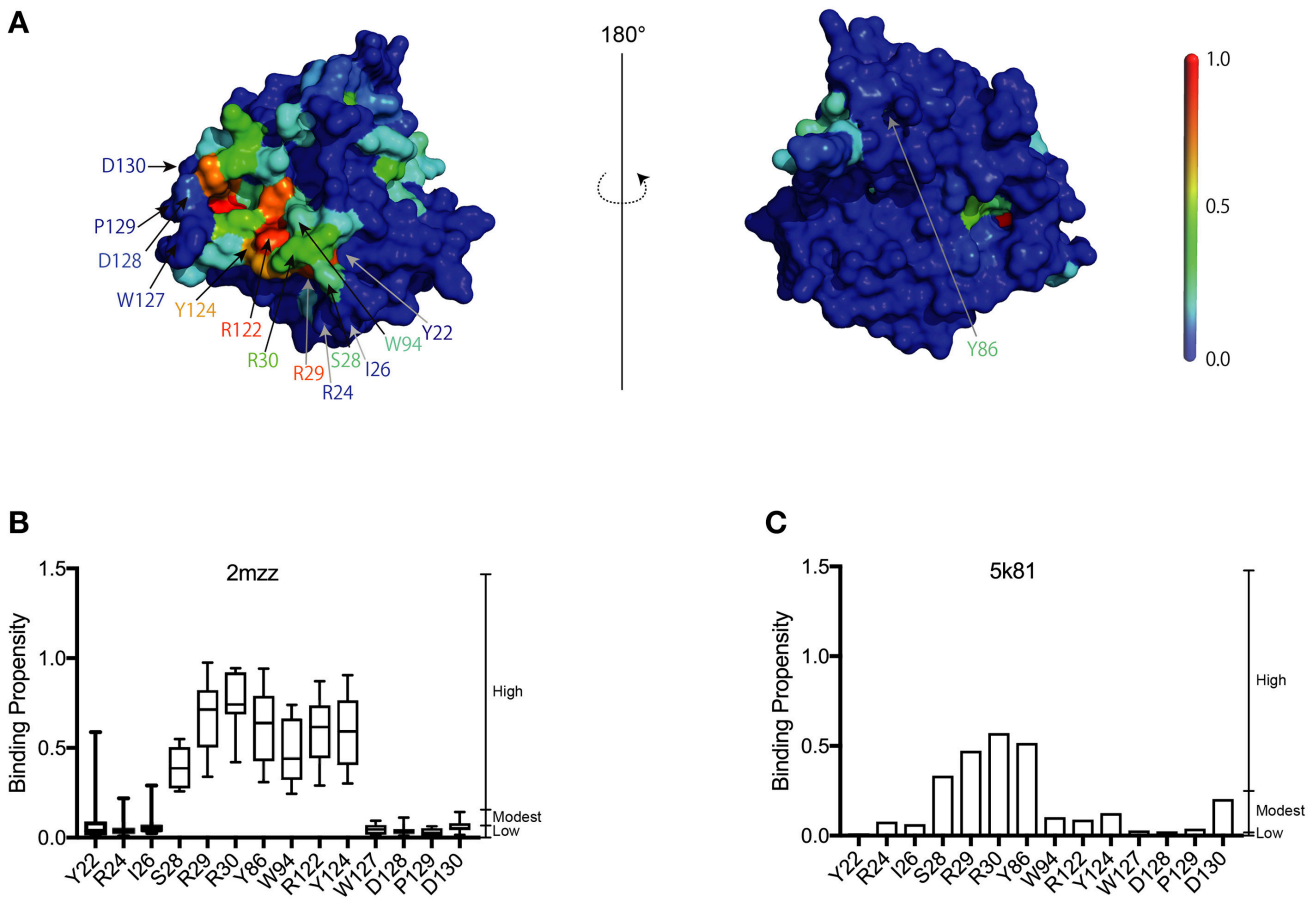
Construction of the A3G-NTD homology model. **(A)** Homology model of wild-type human A3G-NTD based on the soluble A3G-NTD NMR structure (PDB:2mzz) generated by the structural homology modeling program, Spanner. The binding propensities (BP) of residues on the A3G surface are color coded as indicated by the scale bar (high BP, red; low BP blue). The right panel shows the structure model in the left panel rotated 180 degrees. The selected residues that were tested in this study or have been reported to be involved in RNA association or Vif interaction are indicated by arrows. **(B)** RNA-binding propensity (BP) of selected residues from Supplementary Figure 1 predicted by aaRNA. Box plots show the BP distribution for ten models based on soluble A3G NMR structures; error bars represent standard deviation. **(C)** BP of selected residues from Supplementary Figure 2. The bar graph shows the BP of a single model of non-human A3G crystal structure (PDB:5k81).

In the present study, we used a 3D structural model of A3G-NTD WT (1-200) based on the previously published NMR solution structure snapshots of a soluble mutant A3G-NTD (PDBID: 2mzz) (Lis et al., 2011; Kouno et al., 2015) (Figure 1A). In order to consider the flexibility of the protein, we built *in silico* models employing each of the ten NMR structure snapshots available, followed by RNA-binding site predictions generated by the RNA binding site predictor, aaRNA (Li et al., 2014). The aaRNA predictor can generate accurate and robust RNA binding propensities (BP) for each residue based on both sequence and structure features, and is suitable for studying proteins like A3G that have flexible potential binding loops to interact with RNA (Supplementary Figure 1). The aaRNA algorithm predicted residues that show high average RNA BP (Figure 1B and Supplementary Figure 1). We also built another structure model of human A3G-NTD based on the crystal structure of non-human primate A3G-NTD (Xiao et al., 2016) and calculated the BP for each residue (Figure 1C and Supplementary Figure 2).

### Evaluation of the A3G RNA Binding Propensity by Mutational Analysis of the A3G-NTD R122 Residue

**Figure 2:**
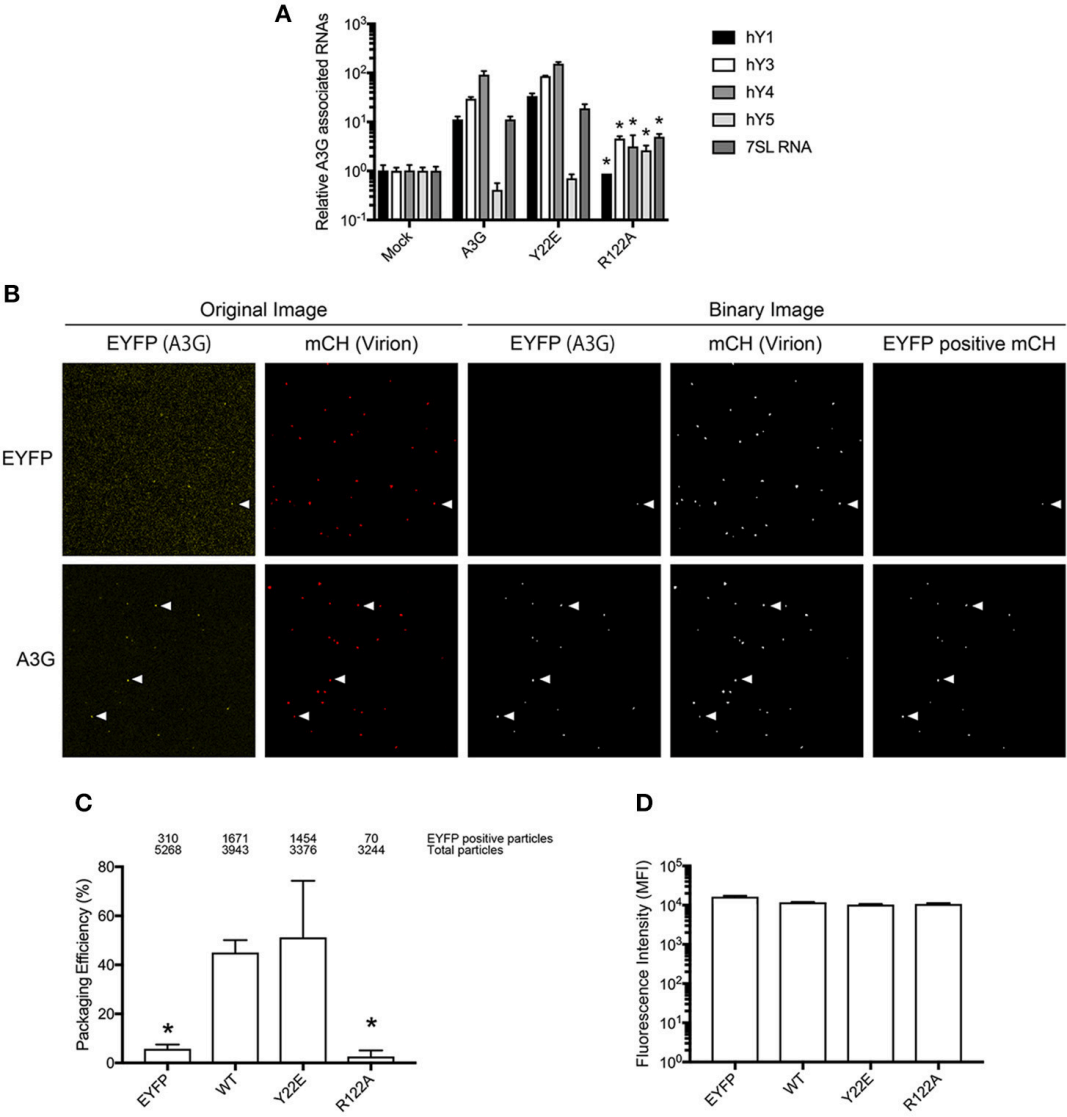
Analysis of the A3G-NTD R122 residue for RNA association and incorporation in HIV-1 virions. **(A)** Selective interaction of A3G and its mutants with cellular RNAs, 7SL and Y RNAs. Analysis of the interactions between cellular RNAs with HA-A3G by qRT-PCR is shown. Cell lysates from transfected HEK293T cells were immunoprecipitated with anti-HA antibody conjugated to agarose beads. RNAs were extracted from the co-immunoprecipitated samples and analyzed by qRT-PCR using primers specific for Y1, Y3, Y4, Y5, and 7SL RNA. Mean values and standard deviations of three independent qPCR reactions are shown for each condition. The level of the mock sample is set to 1. The asterisks indicate statistically significant decreases compared to the amount of cellular RNAs associated with A3G WT calculated by student *t*-test (*p* < 0.05). **(B)** Packaging efficiency of A3G mutants into HIV1-ΔVif virions determined by single virion imaging analysis. Representative images of virus particles labeled with Gag (mCherry) containing EYFP or EYFP-tagged A3G are shown. The left two panels show the original image, and the right three panels show the binary image constructed with an in-house MatLab script. EYFP expression plasmid was used as a negative control. White arrows indicate the mCherry particles that contain EYFP signals. **(C)** Packaging efficiency calculated by the percentage of Gag (mCherry) particles containing EYFP signal. The number of EYFP positive particles (upper) and total Gag-mCherry particles (lower) counted from three independent experiments is shown above the graph. Error bars represent standard deviations for three independent experiments. Asterisks indicate statistically significant decreases compared to EYFP-tagged A3G (student *t*-test, *p* < 0.05). **(D)** Intracellular expression of A3G and its derived mutants in virus producer cells determined by measuring EYFP intensity.

To experimentally validate the *in silico* RNA binding predictions, we generated an *in vitro* mutant of the residue R122. As a positively charged residue, R122 is involved in RNA interactions (Zhang et al., 2010), and is predicted to have a high RNA BP (Figure 1B). As a control, we mutated Y22, which has a low predicted BP (Figures 1B,C). Mutants R122A and Y22E in the full length A3G protein were examined to evaluate the A3G RNA-binding affinity. We immunoprecipitated A3G and performed qRT-PCR with primers to detect the host Y RNAs, previously reported to associate with A3G (Wang et al., 2007). Although all cellular RNAs analyzed, except for hY5, associated strongly with the A3G-WT and the Y22E mutant, the levels associating with the R122A were significantly lower (Figure 2A). We then assessed the efficiency of encapsidation of these A3G mutants into HIV-1-ΔVif virus particles by single virion visualization (Figure 2B) (Hübner et al., 2007; Chen et al., 2009; Burdick et al., 2010; Izumi et al., 2013). A3G-WT and the Y22E mutant were detected in 42.4% and 43.1% of virions, respectively, whereas the R122A mutant was detected in only 4.2% of virions (Figure 2C), confirming previous observations (Huthoff and Malim, 2007). We lysed cells after harvesting the virus supernatant and measured the EYFP fluorescence with a plate reader to verify the cellular expression level of A3G mutants in virus producer cells. The GFP fluorescence intensity has been previously correlated with protein expression of GFP-tagged A3G in producer cells (Matsui et al., 2014). We found that these point mutations did not affect cellular expression of A3G as quantified by their EYFP signal intensities (Figure 2D). These results are consistent with previous reports showing that A3G virion encapsidation is mediated by A3G-RNA association (Luo et al., 2004; Schäfer et al., 2004; Svarovskaia et al., 2004; Zennou et al., 2004).

#### Functional Evaluation of the A3G R122 Mutant

**Figure 3:**
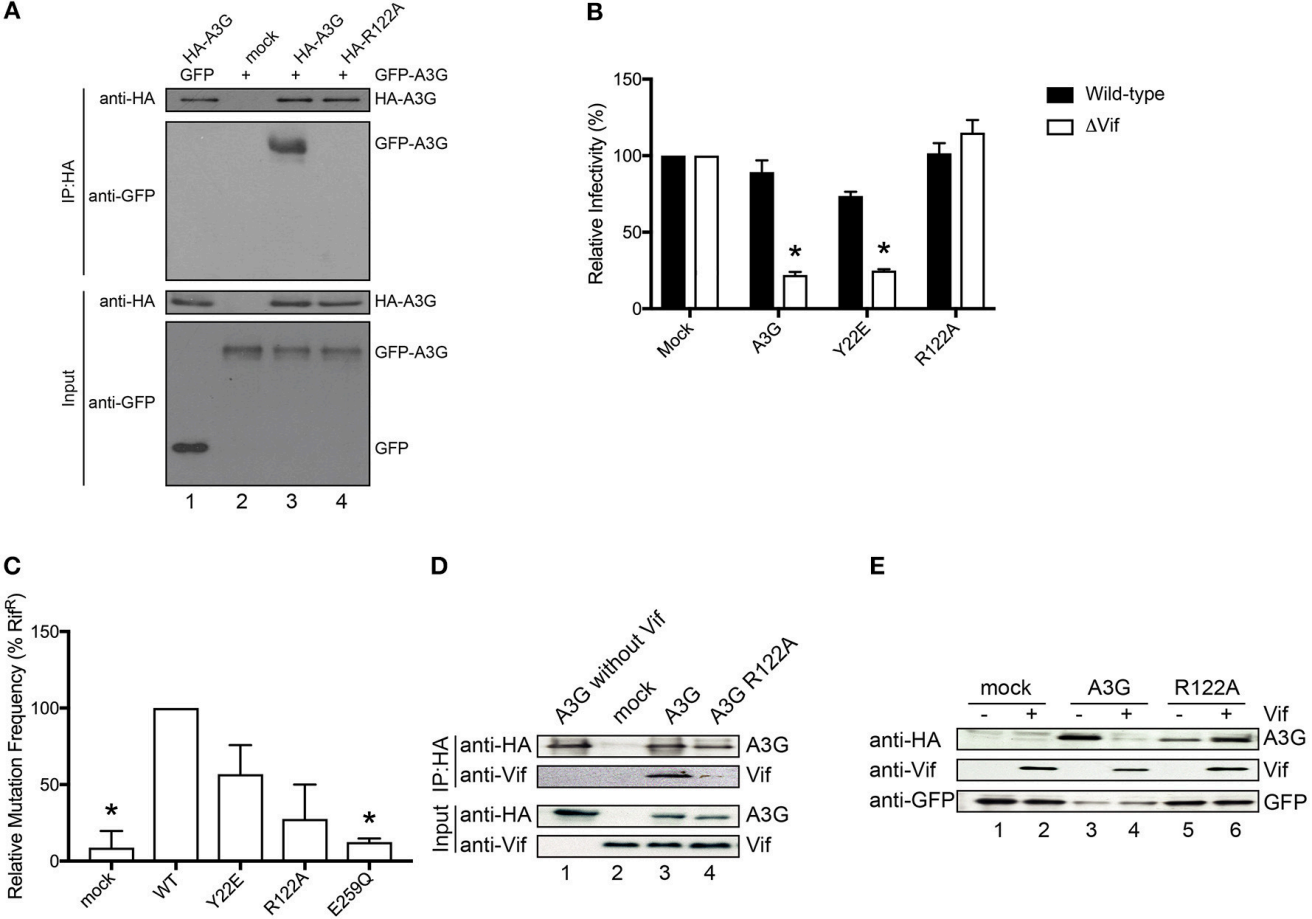
Functional analysis of the A3G-NTD R122 mutant. **(A)** Co-immunoprecipitation of HA-tagged R122A mutant with GFP tagged A3G WT. GFP-tagged A3G and HA-tagged R122A mutant were transiently co-expressed in HEK293T cells. Cell lysate and immunoprecipitated complexes were loaded on SDS-PAGE followed by immunoblot analysis using HA- and GFP-specific antibodies. HA-tagged A3G co-transfected with GFP alone served as a control for nonspecific binding of GFP to HA-A3G (lane 1) and GFP-tagged A3G alone served as a control for nonspecific binding to anti-HA beads (lane 2). The combination of HA-A3G-WT or –R122A mutant with GFP-A3G-WT is shown in lane 3 or lane 4, respectively. **(B)** Single-cycle infectivity of HIV-1 WT or ΔVif viruses produced in the presence of WT or mutant A3G proteins was measured in relative luciferase units and presented as percent infectivity relative to mock. The empty vector control is shown as mock. Three independent experiments were averaged and error bars represent the standard deviations. Asterisks indicate statistical significance of ΔVif virus infectivity compared to WT (student *t*-test *p* < 0.05). **(C)** Editing activity of WT and mutant A3G proteins determined by bacterial mutation assay. A deaminase defective E259Q mutant was used as a negative control. Mock indicates the empty vector control. Error bars represent the standard deviations of two independent experiments. The asterisks indicate statistical significance compared to WT (student *t*-test, *p* < 0.05). **(D)** Co-immunoprecipitation assays to determine the binding of Vif to the A3G-R122A mutant. The expression plasmids for the HA tagged A3G or R122A mutant and Vif were co-transfected into HEK293T cells (lane 3 and lane 4, respectively). MG132 proteasome inhibitor was added 12 h before lysing the cells, and A3G was immunoprecipitated with an anti-HA antibody and analyzed by immunoblot with the indicated antibodies. HA-A3G or Vif expression alone was used as a control for nonspecific binding (lane 1 or lane 2, respectively) **(E)** Vif-dependent degradation of A3G mutant, R122A. We co-transfected the expression vector HA-tagged A3G or R122A mutant with (lane 4 or lane 6, respectively) or without Vif expression plasmid (lane 3 or lane 5, respectively) into HEK293T cells. The amounts of cellular A3G from cells transfected with or without the Vif expressing plasmid were compared by immunoblot assay using an anti-HA antibody. GFP was used as the indicator for transfection efficiency. The lanes without HA-A3G (lane 1 or 2) are controls for nonspecific binding to anti-HA antibody.

It has previously been suggested that the RNA-binding capacity of A3G correlates with its dimerization (Huthoff and Malim, 2007; Chelico et al., 2010; Bélanger et al., 2013). Therefore, to examine the contribution of R122A dimerization to RNA association, we performed co-immunoprecipitation experiments of HA-tagged R122A mutant with GFP tagged A3G WT. We confirmed that the R122A mutant is unable to form an RNA-mediated dimer (Figure 3A and Supplementary Figure 3). We also performed a single-round infection assay to evaluate the antiviral activity of A3G-R122A mutant. We found that A3G WT and the virion-encapsidated mutant, Y22E, inhibited HIV-1-ΔVif infection (Figure 3B). As expected, the R122A mutant, which was unable to dimerize (Figure 3A) or efficiently package into virions (Figure 2C), showed little antiviral activity confirming previous observations (Zhang et al., 2007). We next evaluated the deaminase activity of these A3G mutants by using a bacterial mutation assay. Unlike the E259Q deaminase-defective mutant (Schumacher et al., 2008), the deaminase activities of the Y22E and R122A mutants were not statistically significantly different from the A3G WT deaminase activity (Figure 3C). Altogether, these data showed that the loss of antiviral activity for the R122A mutant was primarily due to its defective virion incorporation. Because the Vif binding region partially overlaps with the RNA interaction domain of A3G-NTD, we further investigated the sensitivity of the R122A mutant toward Vif-mediated degradation. Co-immunoprecipitation of A3G and Vif revealed that the R122A mutation impaired the A3G-Vif interaction (Figure 3D and Supplementary Figure 4). In addition, co-transfection of A3G and Vif without proteasome inhibitor treatment confirmed that A3G-R122A is resistant to Vif-mediated degradation (Figure 3E and Supplementary Figure 5). These data confirmed that residues in the A3G-NTD involved in the interaction with RNA are also involved in the interaction with Vif (Uyttendaele et al., 2012).

#### A3G Binding Propensity to RNA by Single-Virion Analysis

**Figure 4:**
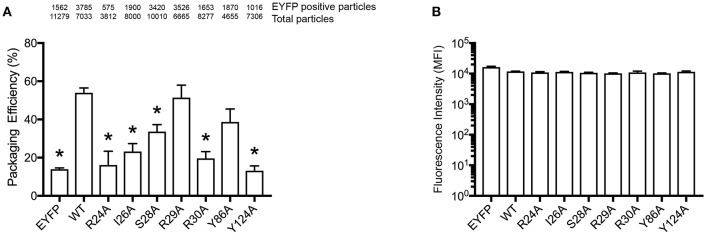
Packaging efficiency of A3G mutants into HIV1-ΔVif virions. **(A)** A3G packaging efficiency, and **(B)** Intracellular EYFP fluorescence intensity are shown as described in the legend for Figures 2C,D. The asterisks indicate statistical significance compared to WT (student *t*-test, *p*<0.05).

In order to confirm our RNA-binding prediction, we further tested the contribution of residues S28, R30, and Y124 that are known determinants of A3G-RNA association (Bulliard et al., 2009; Huthoff et al., 2009; Zhang et al., 2010) and had modest to high BP in our model (Figures 1B,C). In addition to these previously reported RNA-binding residues, we also tested I26, R29, and Y86, which also showed modest to high BP in our prediction (Figures 1B,C). These residues were not previously reported to contribute to RNA association so they were tested in the present study through mutagenesis. We made single alanine substitution mutants in I26, S28, R29, R30, Y86, or Y124 and tested their virion incorporation efficiency. Like R122, mutation of the I26, S28, R30, and Y124 residues significantly disrupted A3G virion incorporation. On the other hand, mutants R29A and Y86A were incorporated into virions to the same extent as A3G-WT (Figure 4A). In our analysis, the BP of the R24 residue was low based on the soluble A3G-NTD homology model (Figure 1B). However, R24 is known to contribute to RNA association (Huthoff et al., 2009). Therefore, we evaluated the virion incorporation efficiency of R24A (Figure 4A) and confirmed that the mutation in this residue impaired A3G virion encapsidation. To control for potential differences in cellular expression of these mutants, we quantified relative expression levels based on EYFP signals and found that there was no difference (Figure 4B).

### Construction of an A3G-NTD RNA Docking Model and Calculation of RNA Contact Frequency

**Figure 5:**
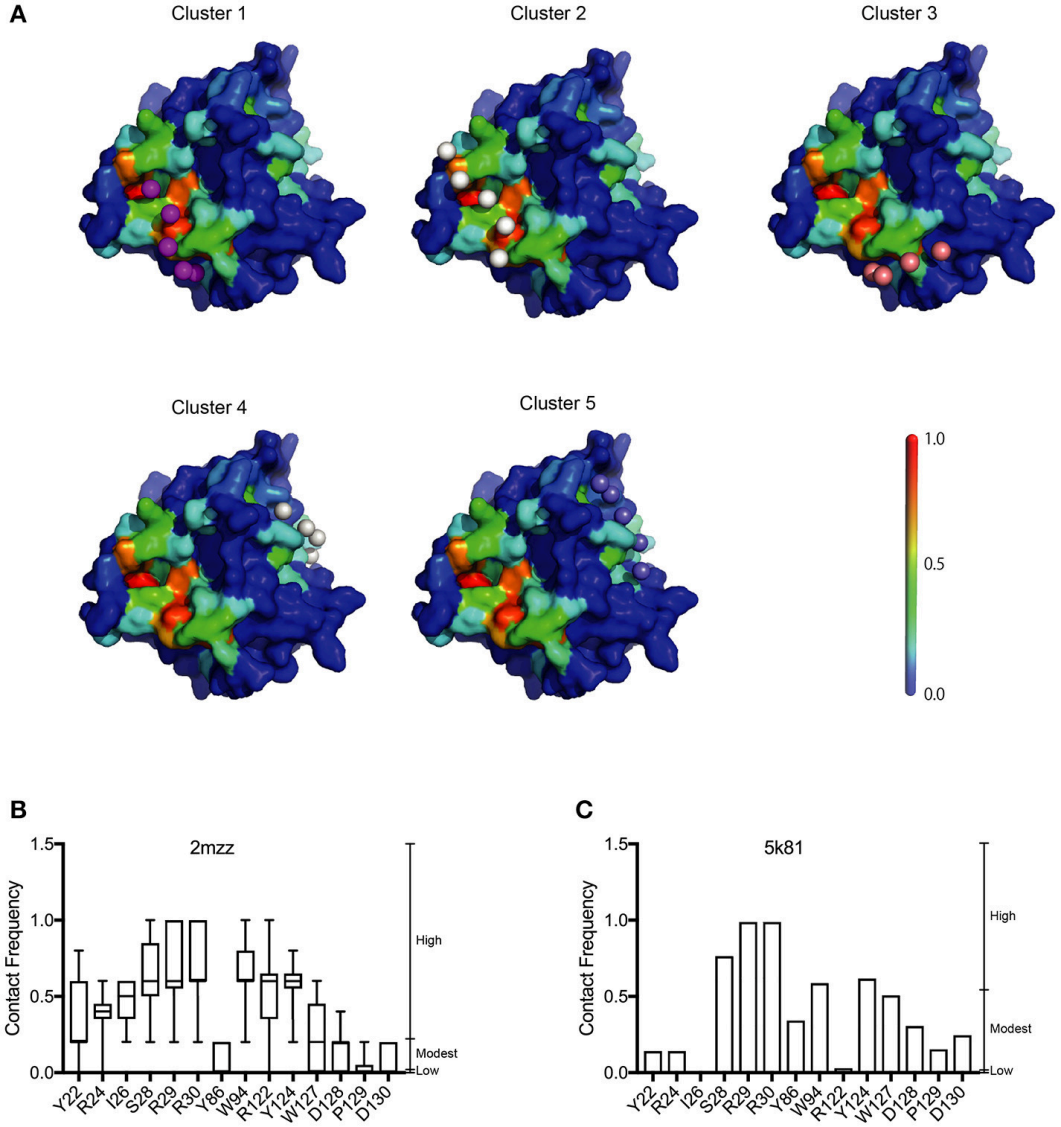
Structural model of the A3G-NTD with single strand RNA docking. **(A)** The top five clusters of docked conformations between the surface of A3G-NTD model based on soluble A3G-NTD and RNA pentamers represented by spheres are shown. The color of the A3G surface shows the BP with the same scale presented in Figure 1A. **(B)** Contact frequency (CF) of selected residues from Supplementary Figure 6 calculated by CGMD docking simulation. Box plots show the mean of CF distribution of ten models of soluble A3G-NTD for the top five clusters. **(C)** CF of selected residues from Supplementary Figure 7. Column bar graph shows CF of a single model of non-human primate A3G-NTD.

Our analysis showed that the BP of A3G to RNA estimated by two-dimensional analysis alone is not sufficient to determine the A3G-NTD residues responsible for RNA-dependent A3G packaging. Because the calculation of the BP scores does not consider geometric relationships, we speculated that additional structural features, such as spatial distance between putative RNA binding residues (high BP residues), the innate A3G-NTD RNA binding surface/bound RNA, and the potential steric hindrance due to amino acids orientation could all determine RNA binding. Therefore, we hypothesized that the potential impact of these factors is minimized on a local scale and RNA contact frequency (CF) can be modeled with the docking of short nucleotides. Recently, York et al. reported about the RNA binding specificity of A3G (York et al., 2016). However, our current computer-assisted docking simulation is limited to using non-specific 5-mer RNA molecules. Therefore, we used an all coarse-grained molecular dynamics (CGMD) docking simulations on the soluble A3G-NTD model with a universal sequence of RNA to identify clusters of binding. We sorted clusters from high to low scores, and the top five were further investigated (Figure 5A). The CF of the top five clusters is summarized in Figure 5B and Supplementary Figure 6. Residue R122, which is spatially close to W94, mediated direct association with RNA. S28, R30, and Y124, constituted a cluster involved in RNA interaction. R24 and I26 located beside R30 also had high-predicted CF (Figure 5B). On the other hand, both BP and CF values for R29 were similar to R30 (Figures 1B, 5B). Interestingly, mutation of R29 did not impede A3G virion encapsidation (Figure 4A). Because of the proximity of residue R30 to R29, we could not examine the function of these residues on the basis of the *in silico* simulations alone, so more detailed experimental analyses will be needed. Residue Y86 located on the opposite side of the RNA-associating surface (Figure 1A) had moderate CF (Figure 5B), suggesting a minor role in the formation of the RNA-A3G-NTD complex. We also calculated the CF of each residue on the non-human primate model, and most residues showed similar patterns to the soluble form model (Figure 5C and Supplemental Figure 7). However, the RNA associating residues R24, I26, and R122 had lower CF in this model (Figure 5C), suggesting that the soluble A3G-NTD model provides better structural predictions of RNA association. I26 has been reported to be involved in Vif-interaction and A3G dimerization (Gorle et al., 2017; Zhai et al., 2017), but it has not been identified as an RNA associating residue. Both R24 and I26 RNA BPs were predicted to be modest (Figure 1B). However, these residues are located in the A3G dimerization surface, which is important for A3G-RNA interaction, suggesting that they may indirectly contribute to RNA association. Taken together, residues R24, I26, S28, R30, R122, and Y124 are involved in the interaction with RNA, actively supporting our A3G-RNA docking model.

## Discussion

Here, we report the utilization of *in silico* approaches (RNA binding propensity prediction, flexible RNA docking and structural modeling) to identify putative RNA-binding residues in the N-terminal domain (NTD) of A3G. Our model identified the previously reported RNA-associating hot-spot residues, S28, R30, R122, and Y124 and we confirmed their involvement in RNA binding in a series of mutagenesis experiments. Interestingly, our experimental data confirmed that the I26 residue is an RNA binding determinant as initially predicted in our model. Although I26 is a known determinant of A3G-Vif interactions (Gorle et al., 2017), here we report for the first time that I26 is also involved in RNA binding. In addition to I26, we also confirmed that the nearby residue R24, which was previously reported by others to be involved in the association with several A3G interacting RNAs (Table 1), mediates RNA interaction (Figure 4A) despite a low BP in our simulation (Figure 1B). These examples suggest the importance of flexible RNA docking in addition to BP prediction. Residues with low BP but located near high-BP regions can act as bridges to facilitate protein-RNA interactions. Moreover, these residues are located in the A3G-dimerization surface and their mutation impairs dimerization and RNA association (Gorle et al., 2017; Zhai et al., 2017). On the other hand, residue Y86, which showed high BP (Figure 1B), was located on the opposite side of the RNA-interaction surface (Figures 1A, 5A) and had moderate CF (Figure 5B). Experimentally, Y86 did not appear to be involved in RNA association (Figure 4A). These data highlight an essential aspect of the RNA-association prediction: residues with low to moderate BP, but high CF and located near high BP residues can also be determinants of RNA binding.

Recently Polevoda et al. investigated the competitive binding of RNA and single-strand DNA (ssDNA) to A3G (Polevoda et al., 2015). They isolated a number of peptides in the A3G domain that bind to RNA, solely or in competition with ssDNA. They used a cross-linking method to show that non-specific 25nt RNA binds to A3G in both the N- and C-terminal domains. The peptides that stood out within the NTD were aa 15–29, 41–52, 83–99, and 181–194 with only the last being a ssDNA binding site. In our study, the RNA BP of most residues corresponding to these peptides was moderate to low (Figure 1B and Supplementary Figure 1). However, some of the residues with high BP or CF overlapped: 15–29 contains S28 and 83–99 contains W94 (Figures 1B, 5B). Interestingly, all of these residues were located on the surface of the RNA binding region in our model (Figure 5A) and showed high CF (Supplementary Figure 6). The peptide corresponding to residues 181–194 had a low BP but modestly high CF. On the other hand, the peptide corresponding to residue 41–52, located on the opposite side of the RNA binding surface, showed both low BP and CF in our simulations. We speculate that these discrepancies are due to the increase in conformational flexibility of the peptides along with the influence of spatially proximate residues in the docking simulations.

The loss of the A3G-NTD results in a drastic decrease in DNA binding affinity, although the C-terminal domain of A3G is responsible for its catalytic activity, and the A3G-CTD alone is able to bind and deaminate ssDNA (Holden et al., 2008). A3G-NTD is also considered important for ssDNA interaction and can greatly enhance the deamination efficiency (Holden et al., 2008; Chelico et al., 2010). Therefore, we additionally calculated the BP of each residue in our A3G-NTD model and performed the A3G-ssDNA docking to estimate CF (Supplementary Figures 8, 9). The distribution of both BP and CF for ssDNA binding showed a similar pattern to the ssRNA association (Table 1), indicating that ssDNA and ssRNA may interact with the same surface of A3G-NTD. Together, these data suggest that the conservation of RNA binding affinity in the A3G-NTD indirectly contributes to A3G's HIV-1 ssDNA deamination activity.

In order to examine the validity of our predictions, we built a second structural model of the human A3G-NTD based on the crystal structure of non-human primate A3G-NTD (Xiao et al., 2016) and performed similar RNA docking simulations. Generally, the BP and CF profiles of the non-human primate crystallography model and soluble human A3G model were similar (Supplementary Figures 1, 2, 6, 7). However, Y86, but not I26, R24, and R122 were predicted to be involved in RNA association in their CFs (Figure 5C), but were not experimentally validated (Figure 4A). These data suggest that the soluble model is a better template for RNA docking simulation. In this regard, the template of the soluble model has been recently revealed by NMR and contains ten snapshots, whereas the non-human primate model derives from one crystal structure and therefore has little protein flexibility. Our aaRNA prediction considers accessibility to the residue: the lower the accessibility, the less the binding likelihood. Certain RNA binding residues could be more accessible in some of the soluble A3G models, but inaccessible in the single crystal structure model. This may explain the overall better agreement of the soluble model predictions with the experimental observations. In addition, the soluble human A3G-NTD has 80% sequence identity to the wild-type human A3G. On the other hand, though the non-human primate A3G has 69% sequence identity to the wild-type human A3G-NTD, the lower sequence identity could account for some of the experimental discrepancies. The root-mean-squares-deviation (RMSD) between the soluble and the non-human primate model was 3.595, indicating not a drastic but significant difference between these two models. Taken together, the soluble A3G-NTD model provided the highest accuracy of RNA association and further refinements of the non-human primate model are desirable. Nevertheless, all of the RNA associating residues that we confirmed experimentally are highly conserved in human and non-human primate A3Gs (Chimpanzee, Gorilla, Orangutan, Macaque, and African green monkey) (Zhang and Webb, 2004). This suggests that the RNA associating surface in the A3G-NTD is highly conserved across species.

To examine our findings in the context of the state of the art, we confirmed that residues R122, predicted here to be involved in the interaction with RNA (Figure 2A) have also been shown to interact with Vif (Figures 3D,E). Interestingly, D128 and its surrounding residues, P129 and D130, critical determinants of the species-specific sensitivity of A3G to Vif, but do not contribute to virion encapsidation (Bogerd et al., 2004; Mangeat et al., 2004; Schrofelbauer et al., 2004; Xu et al., 2004), had modest to low BP and CF in our model (Figures 1B, 5B). Together, the previous reports (Bogerd et al., 2004; Mangeat et al., 2004; Schrofelbauer et al., 2004; Xu et al., 2004; Zhang et al., 2010; Letko et al., 2015) and our R122 mutation assay suggest that the Vif-interacting region of A3G-NTD is close, but distinct from the RNA-binding interface. Therefore, our work provides insights to better understanding the structural nuances of A3G-RNA and Vif binding, subsequently could aid the *in silico* design of small molecules that specifically target the A3G-Vif complex without disrupting the A3G-RNA binding.

Interestingly, it has been reported that the Vif mediated A3G inhibition is not only mediated by proteosomal degradation but also by translational repression (Stopak et al., 2003; Guerrero et al., 2016). Though it remains unclear how important each of these pathways are relative to one another *in vivo*, these findings suggested that both pathways could be targeted to restore cellular A3G levels. Inhibiting both pathways could potentially lead to a more effective, multipronged approach to restoring A3G cellular levels for packaging. Interestingly, Guerrero et al. demonstrated that A3G degradation deficient mutant, K26R was still able to decrease A3G mRNA expression, and concluded that these two pathways are independent. On the other hand, they also showed that mutants of certain A3G-binding residues in Vif, H42 and H43, did not also inhibit A3G translation, suggesting that the residues in Vif responsible for translational repression may be partially located in the A3G-Vif interacting domain, or this function may require A3G-Vif interaction. In either case, A3G-Vif interaction inhibitors could potentially block both of Vif's A3G inhibitory pathways. In addition, Letko et al. simulated a docking model of A3G-NTD and Vif, indicating that A3G β4-α3 loop composed of Y125, D128, and D130 fitted in the Vif pocket (Letko et al., 2015). In their model, the Vif residues at position 14–17, 19, and 22 formed one side of the pocket, and the other side flanked by the residues at 40–44 responsible for A3G translational repression. According to their docking model, it would be possible to design small molecules that inhibit both Vif activities against A3G protein expression and stability.

Naturally occurring non-synonymous changes in the A3G interaction domains are of clinical importance and could alter RNA binding. In this regard, Matume et al. recently reported four non-synonymous polymorphisms (H186R, R256H, Q275E, and G363R) in the A3G genes in a cohort of HIV-1 infected South African individuals (Matume et al., 2019). Of these residues, only H186 is present in the A3G-NTD. With our *in silico* model, we found that the residue at position 186 had a low CF score (Supplementary Figure 6), indicating that it may be less relevant to RNA association. Future clinical investigations may reveal other A3G polymorphisms important for the RNA association.

In summary, we applied a novel approach using *in silico* models to predict A3G-RNA interaction sites and experimentally confirmed these predictions. In addition, we utilized the models to identify a novel residue, I26 that contributes to RNA association. Together with previous alanine scanning experiments and structural modeling of Vif docking to A3G, the RNA interaction predictions described here could be used for *in silico* drug screening or design of selective inhibitors of A3G-Vif binding while maintaining the A3G-RNA association necessary for virion incorporation.

## Data Availability

All datasets generated for this study are included in the manuscript and/or the Supplementary Files.

## Author Contributions

HF, LS, AS, and TI performed the experiments. SL, KY, DS, and TI designed and analyzed the data. LS, JS, KS, AT-K, and TI wrote the manuscript. KS, AT-K, and TI contributed financial assistance.

### Conflict of Interest Statement

The authors declare that the research was conducted in the absence of any commercial or financial relationships that could be construed as a potential conflict of interest.

## Supplementary Material

Supplementary Figure 1 RNA-binding propensity (BP) of all residues in the homology model of human A3G-NTD based on the solution NMR human A3G-NTD structure (PDBID: 2mzz) predicted by aaRNA. The box plot shows the distribution of the BP for ten structures.

Supplementary Figure 2 RNA-binding propensity (BP) of all residues in the homology model of human A3G-NTD simulated based on the crystal structure of a non-human primate A3G (PDBID: 5k81).

Supplementary Figure 3 Raw images of western blot analysis presented in Figure 3 are shown.

Supplementary Figure 4 Raw images of western blot analysis presented in Figure 3D are shown.

Supplementary Figure 5 Raw images of western blot analysis presented in Figure 3E are shown.

Supplementary Figure 6 RNA-contact frequency (CF) of all residues in the homology model of human A3G-NTD based on the human A3G-NTD solution NMR structure (PDBID: 2mzz) with 5-mer single strand RNAs calculated by CGMD docking simulation. The box plots show the distribution of the CF of ten models with the top five clusters.

Supplementary Figure 7 RNA-contact frequency (CF) of all residues in the homology model of human A3G-NTD simulated based on the crystal structure of a non-human primate A3G (PDBID: 5k81). The column bar graph shows the mean of the CF of a single model with the top five clusters.

Supplementary Figure 8 DNA-binding propensity (BP) of all residues in the homology model of human A3G-NTD based on the human A3G-NTD solution NMR structure (PDBID: 2mzz) predicted by aaDNA. The box plot shows the distribution of the BP for ten structures.

Supplementary Figure 9 DNA-contact frequency (CF) of all residues in the homology model of human A3G-NTD based on the human A3G-NTD solution NMR structure (PDBID: 2mzz) with 5-mer single strand DNAs calculated by CGMD docking simulation. The box plots show the distribution of the CF of ten models with the top five clusters.
