# Supplementary figures and images for: Structural Determinants of the APOBEC3G N-Terminal Domain for HIV-1 RNA Association
Source: Front Cell Infect Microbiol. 2019 May 21;9:129. doi: 10.3389/fcimb.2019.00129 (PMC6536580; doi:10.3389/fcimb.2019.00129)

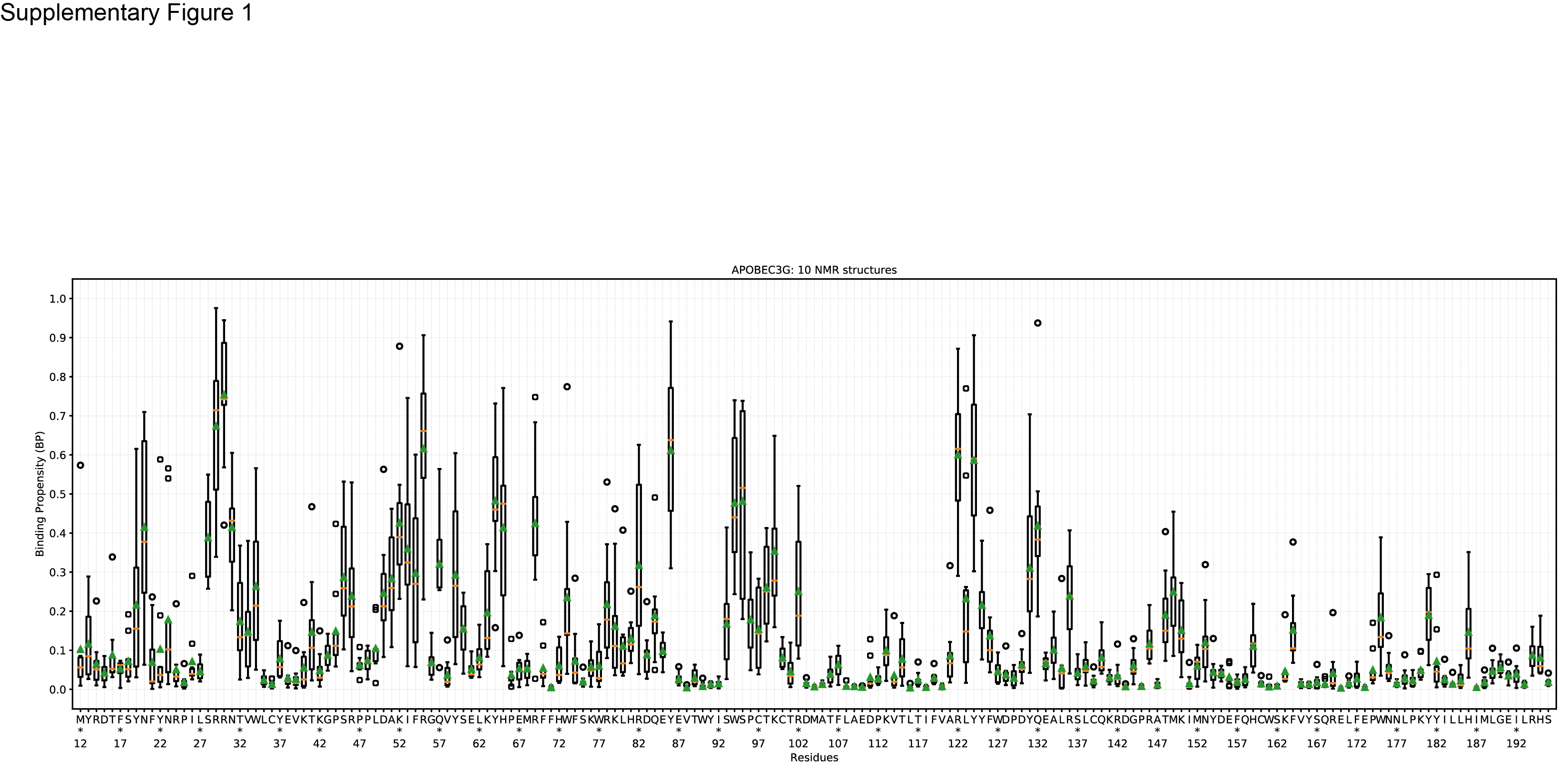

Supplement: Supplementary Figure 1 — RNA-binding propensity (BP) of all residues in the homology model of human A3G-NTD based on the solution NMR human A3G-NTD structure (PDBID: 2mzz) predicted by aaRNA. The box plot shows the distribution of the BP for ten structures. [file Image_1.JPEG]

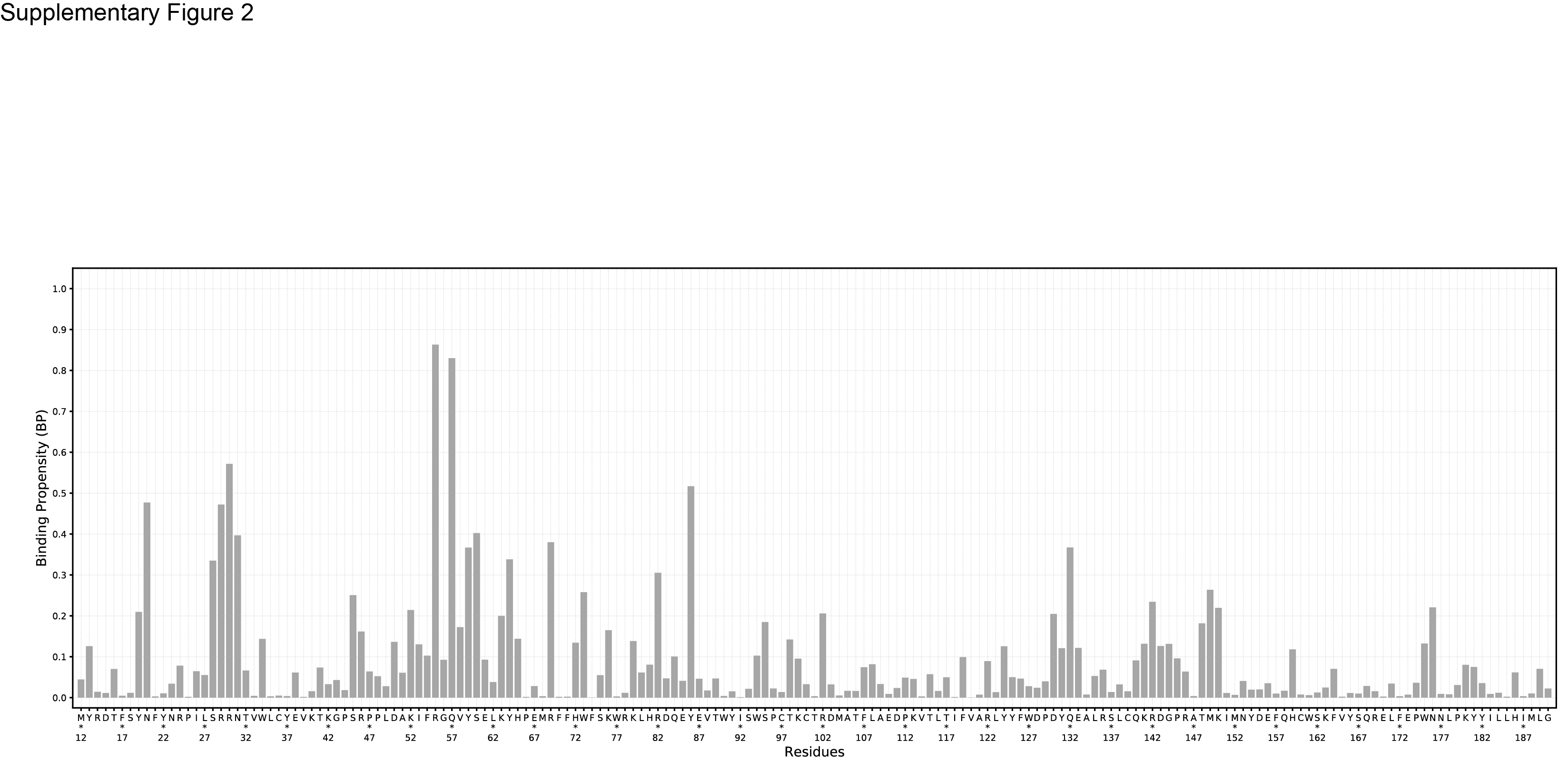

Supplement: Supplementary Figure 2 — RNA-binding propensity (BP) of all residues in the homology model of human A3G-NTD simulated based on the crystal structure of a non-human primate A3G (PDBID: 5k81). [file Image_2.JPEG]

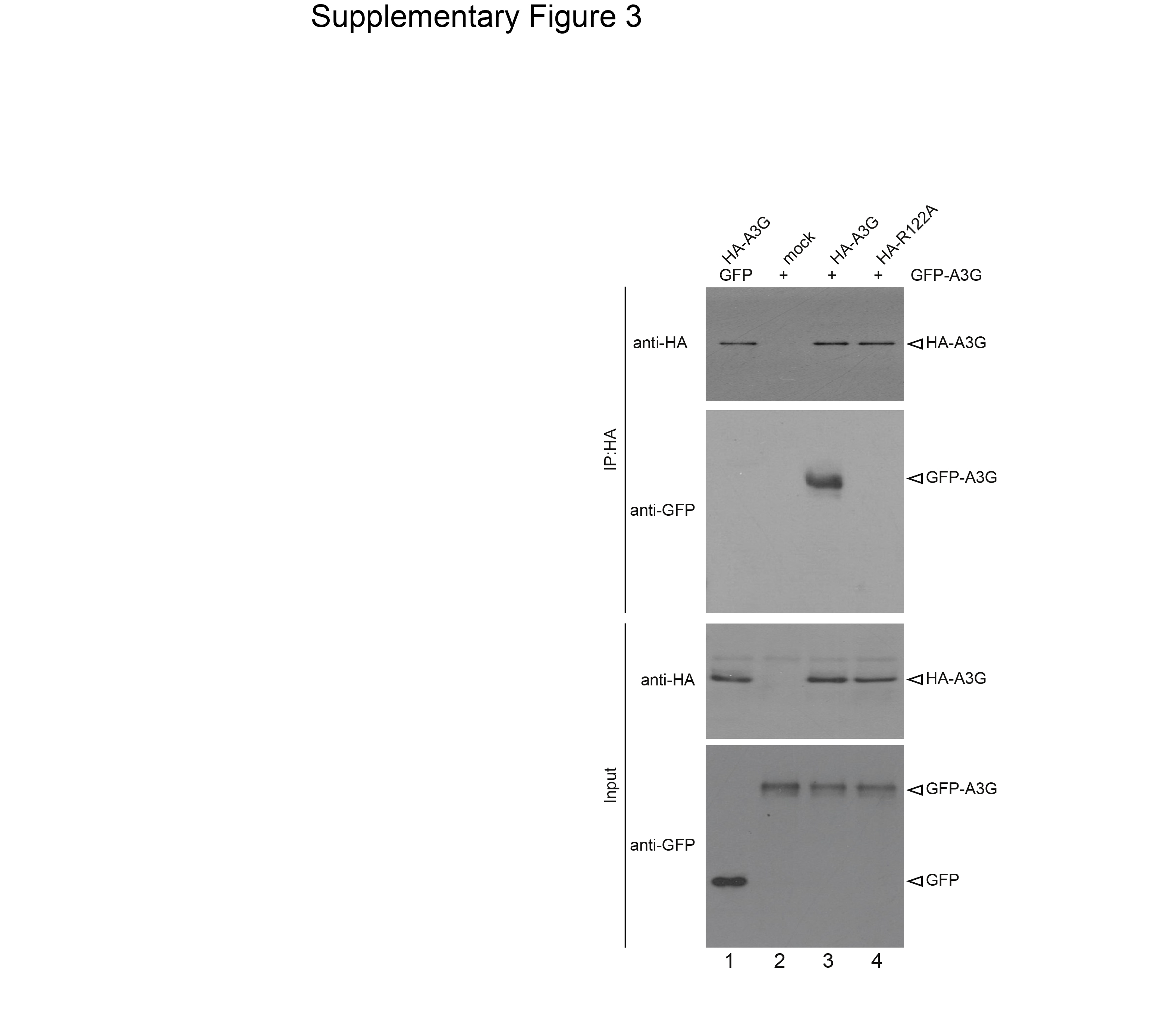

Supplement: Supplementary Figure 3 — Raw images of western blot analysis presented in Figure 3 are shown. [file Image_3.jpg]

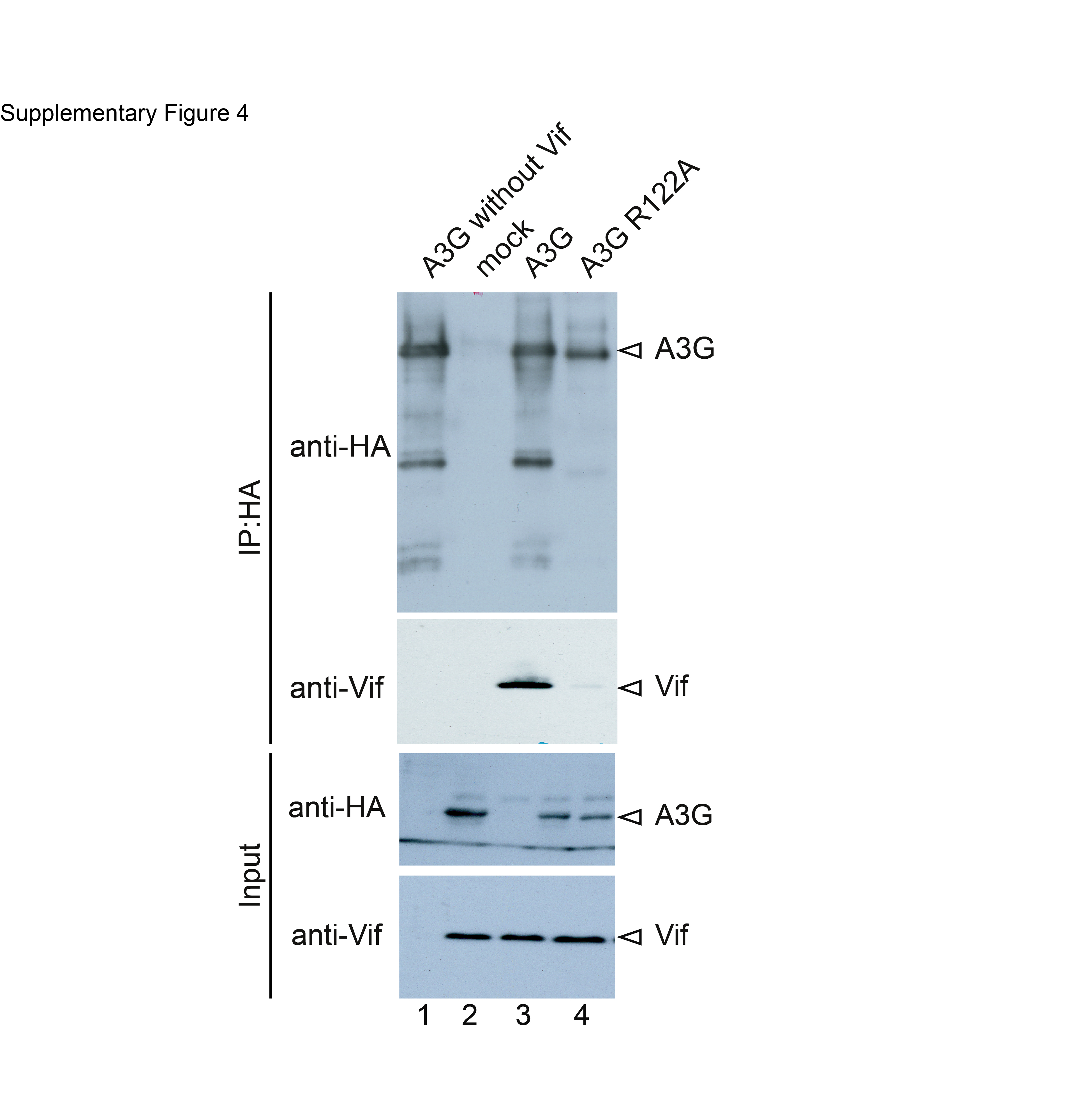

Supplement: Supplementary Figure 4 — Raw images of western blot analysis presented in Figure 3D are shown. [file Image_4.jpg]

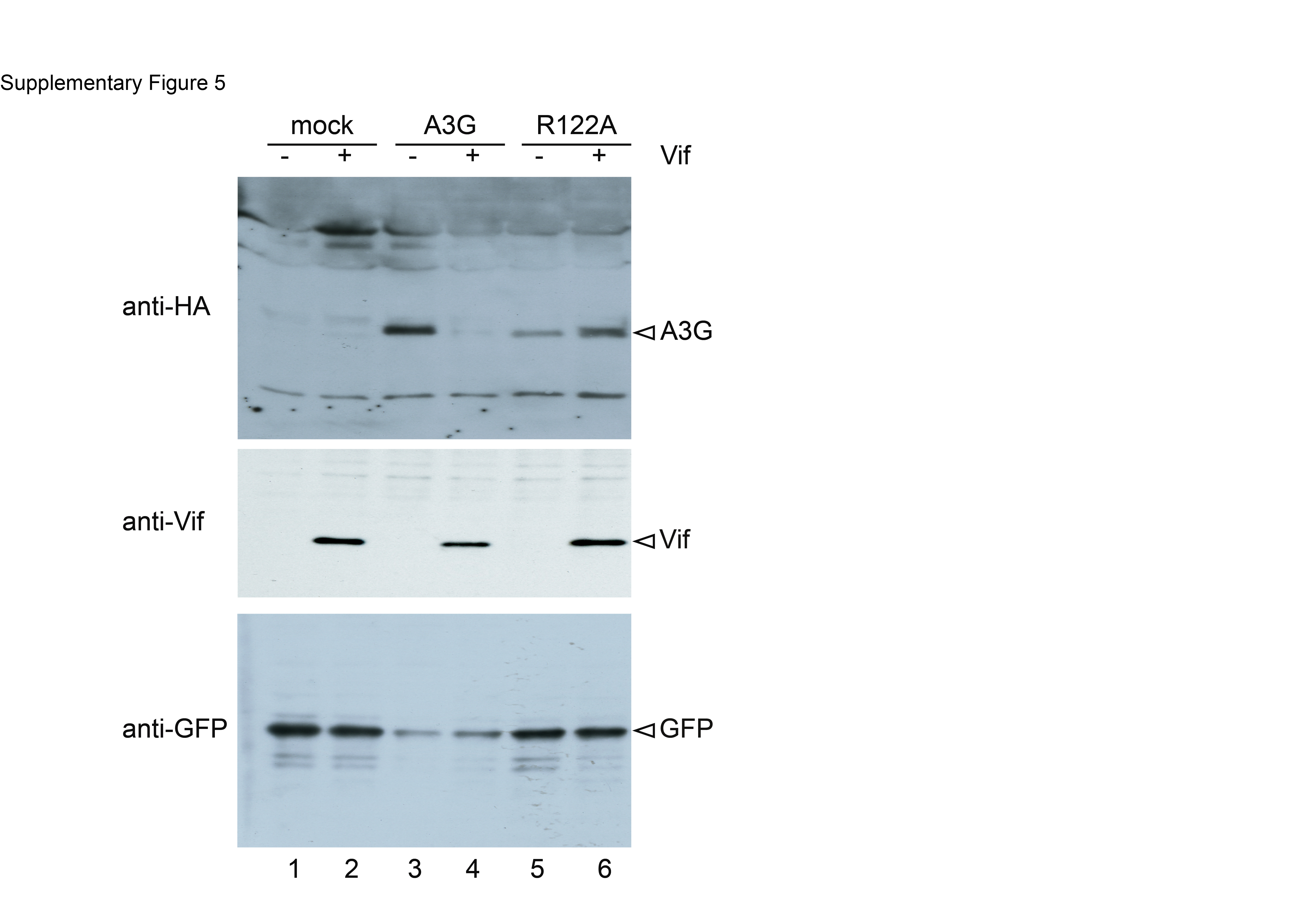

Supplement: Supplementary Figure 5 — Raw images of western blot analysis presented in Figure 3E are shown. [file Image_5.jpg]

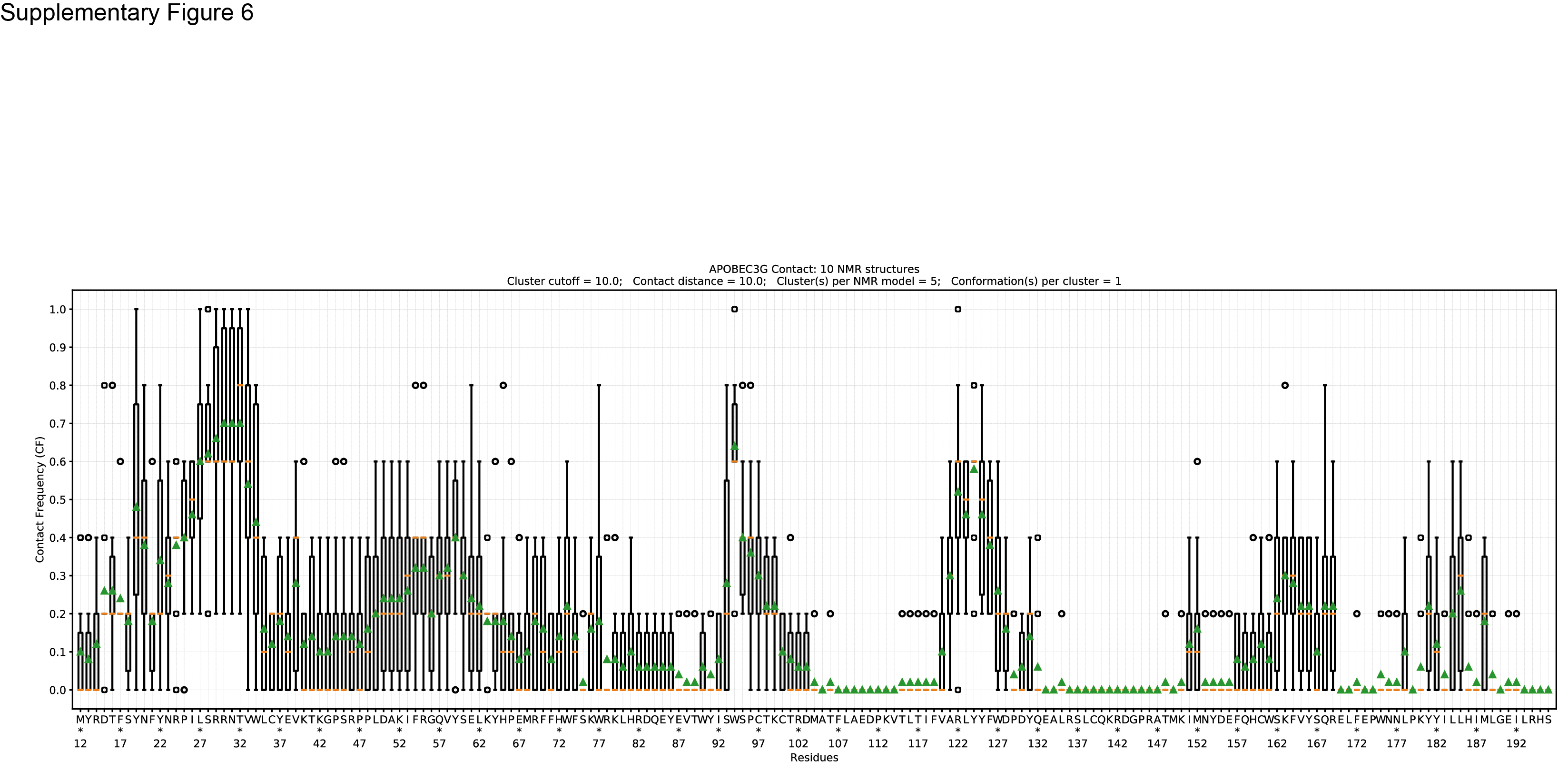

Supplement: Supplementary Figure 6 — RNA-contact frequency (CF) of all residues in the homology model of human A3G-NTD based on the human A3G-NTD solution NMR structure (PDBID: 2mzz) with 5-mer single strand RNAs calculated by CGMD docking simulation. The box plots show the distribution of the CF of ten models with the top five clusters. [file Image_6.JPEG]

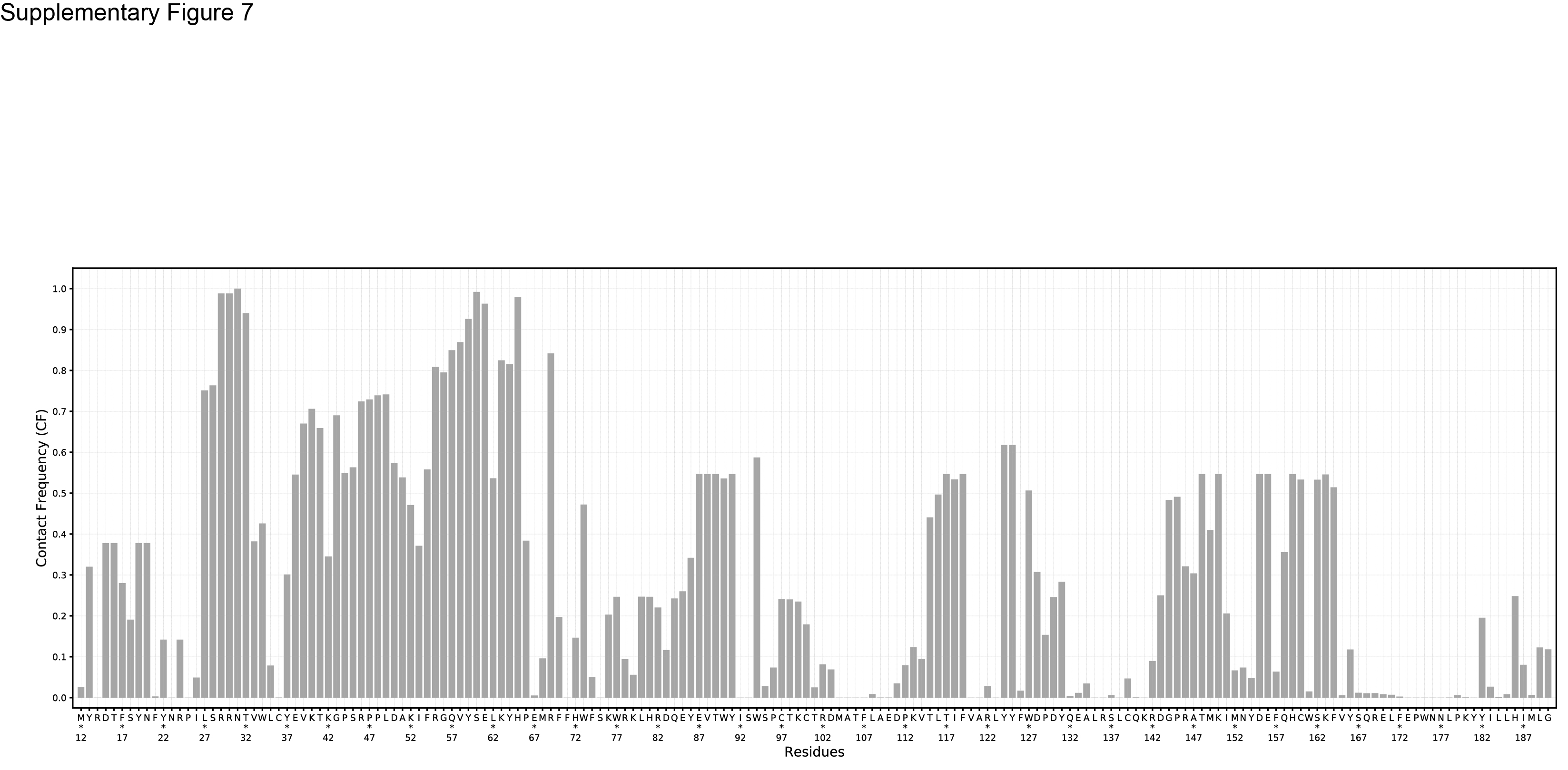

Supplement: Supplementary Figure 7 — RNA-contact frequency (CF) of all residues in the homology model of human A3G-NTD simulated based on the crystal structure of a non-human primate A3G (PDBID: 5k81). The column bar graph shows the mean of the CF of a single model with the top five clusters. [file Image_7.JPEG]

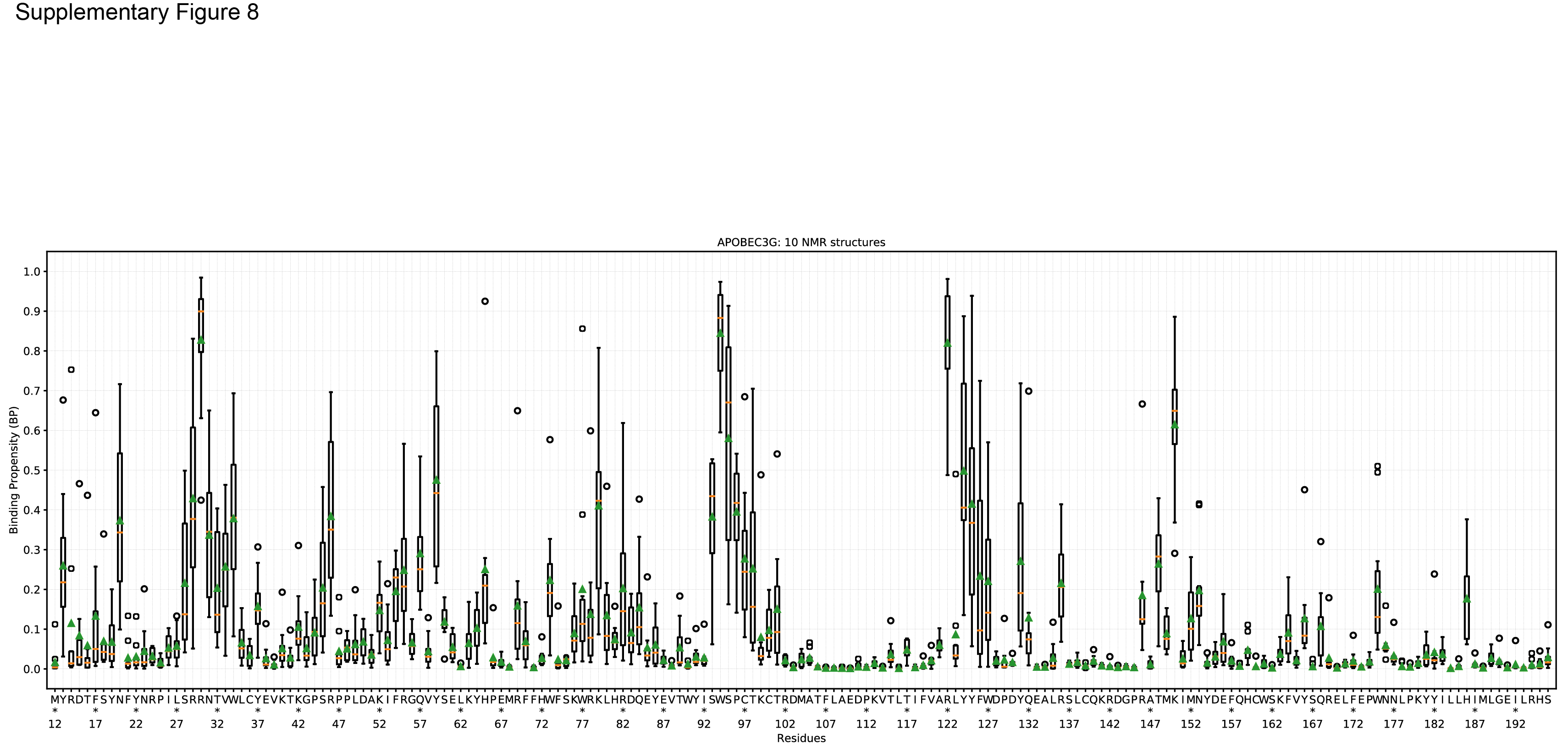

Supplement: Supplementary Figure 8 — DNA-binding propensity (BP) of all residues in the homology model of human A3G-NTD based on the human A3G-NTD solution NMR structure (PDBID: 2mzz) predicted by aaDNA. The box plot shows the distribution of the BP for ten structures. [file Image_8.JPEG]

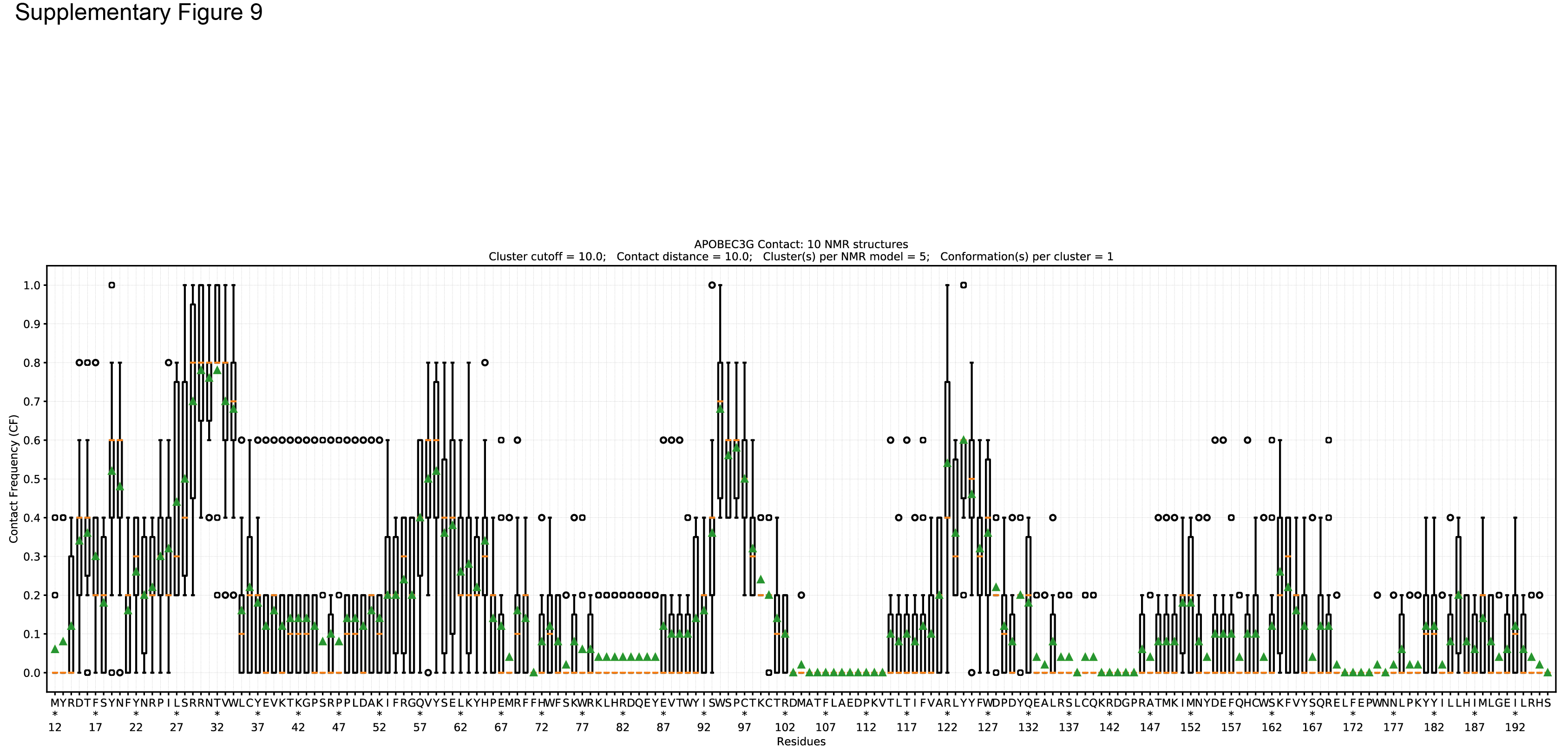

Supplement: Supplementary Figure 9 — DNA-contact frequency (CF) of all residues in the homology model of human A3G-NTD based on the human A3G-NTD solution NMR structure (PDBID: 2mzz) with 5-mer single strand DNAs calculated by CGMD docking simulation. The box plots show the distribution of the CF of ten models with the top five clusters. [file Image_9.JPEG]
